# Famciclovir (FAMVIR^®^)

**DOI:** 10.1155/S1064744997000033

**Published:** 1997

**Authors:** Stephen L. Sacks

**Affiliations:** 1 Department of Pharmacology and Therapeutics Faculty of Medicine The University of British Columbia British Columbia Vancouver Canada; 2 Viridae Clinical Sciences, Inc. 1134 Burrard Street British Columbia Vancouver V6Z 1Y8 Canada

## Abstract

Famciclovir is an antiviral with efficacy and safety comparable to aciclovir, but famciclovir's more favorable pharmacokinetic profile enables a less frequent dosing regimen. Future trials will likely determine famciclovir's role in the suppression of HBV.


Infectious Diseases in Obstetrics and Gynecology 5:3–7 (1997)
© 1997 Wiley-Liss, Inc.

### Famciclovir (FAMVIR®)

**Stephen L. Sacks***
*Department of Pharmacology and Therapeutics, Faculty of Medicine, The University of British Columbia, Vancouver, British Columbia, Canada*
*Viridae Clinical Sciences, Inc., Vancouver, British Columbia, Canada*

Abstract

**Famciclovir is an antiviral with efficacy and safety comparable to aciclovir, but famciclovir's more favorable pharmacokinetic profile enables a less frequent dosing regimen. Future trials will likely determine famciclovir's role in the suppression of HBV. Infect. Dis. Obstet. Gynecol, 5:3–7, 1997.**
© 1997 Wiley-Liss, Inc.

Famciclovir (Famvir®, SmithKline Beecham, Crawley, UK) is a prodrug of the antiviral nucleoside analog penciclovir. It is currently approved worldwide for the treatment of varicella zoster (VCV) and herpes simplex virus (HSV-1 and HSV-2) infections. Famciclovir is characterized by its high rate of absorption and rapid conversion to the active compound, penciclovir. In the U.S., the currently approved dosing regimen of famciclovir for the treatment of herpes zoster (shingles) is 500 mg tid for 7 days; for recurrent genital herpes it is 125 mg bid for 5 days. This treatment is also effective for treatment of first episode genital herpes at a dose of 250 mg tid for 5 days and chronic suppression of recurrences at a dose of 250 mg bid. However, the last two indications are still pending approval by U.S. regulatory authorities.

### STRUCTURE AND DERIVATION

Famciclovir (2-[2-(2-amino-9H-purin-9-yl)ethyl]-1,3-propanediol diacetate) is the diacetyl ester of 6-deoxy penciclovir (a synthetic acyclic guanine derivative). Whereas penciclovir itself is very poorly absorbed orally, it was found in animal studies that its dipropionyl and diacetyl 6-deoxy derivatives (BRL 43599 and famciclovir, respectively) gave much improved blood levels of penciclovir. Furthermore, famciclovir was found to be much more stable than BRL 43599.[1] Human studies confirmed that, after oral dosing with famciclovir, more than half the dose is absorbed.

### MECHANISM OF ACTION

Penciclovir freely enters both virus-infected and uninfected cells. However, antiherpesviral selectivity relies on rapid phosphorylation to the active form, penciclovir triphosphate, in virus-infected cells only. Viral thymidine kinase (TK) is responsible for the critical rate-limiting initial phosphorylation to penciclovir monophosphate. Metabolism to the diphosphate and active triphosphate forms occur via other cellular enzymes.[2]

Penciclovir triphosphate accumulates to high concentrations in virus-infected cells and interacts with viral DNA polymerase to inhibit viral DNA synthesis.[3] Although penciclovir is phosphorylated in uninfected cells as well, conversion to penciclovir monophosphate is much more readily achieved by viral than by human TK. Thus, concentrations of penciclovir triphosphate remain low in uninfected cells and have minimal effect on human DNA.[2]

### PHARMACOKINETICS

After oral administration, famciclovir undergoes rapid and extensive absorption in the upper intestine and is rapidly metabolized in the intestinal wall and liver to the active compound penciclo-

*Correspondence: Dr. Stephen L. Sacks, Viridae Clinical Sciences, Inc., 1134 Burrard Street, Vancouver, B.C. V6Z 1Y8, Canada.

Received 15 November 1996
Accepted 14 January 1997

vir.[4,5] Little or no famciclovir is detected in plasma or urine. Inactive metabolites formed include 6-deoxy penciclovir, monoacetylated penciclovir, and 6-deoxy monoacetylated penciclovir.[6] Unlike aciclovir, which has a 10–20% bioavailability,[7] the bioavailability of penciclovir after oral administration of famciclovir is about 77%.[4,8,9] Compared to aciclovir, penciclovir is preferentially taken up and phosphorylated by HSV- and VZV-infected cells. Furthermore, penciclovir triphosphate has a very prolonged intracellular half-life in infected cells (7 to 20 hours versus 0.7 to 1 hour for aciclovir triphosphate).[2] Penciclovir is not metabolized but is eliminated unchanged in the urine.[10]

The pharmacokinetics of penciclovir remain largely unaltered in the elderly, making it unnecessary to adjust the dosage in these patients.[11,12]

In a study of patients with renal impairment, urinary excretion of penciclovir decreased in proportion to the decrease in estimated creatine clearance leading to a recommendation that the dosage interval of famciclovir should be prolonged from 8 hours to 12 hours for patients with moderate renal impairment, and to 24 hours for patients with severe renal impairment.[13] There is no evidence for the need to alter dosage levels of famciclovir in those with well-compensated hepatic impairment.[14] The pharmacokinetics of famciclovir have not been studied in patients with severe uncompensated hepatic impairment.

In three studies that investigated the effects of food on the pharmacokinetics and bioavailability of famciclovir,[15]–17 no clinically significant differences were observed in healthy male volunteers who received famciclovir (250 and 500 mg single doses) 30 minutes after food compared to those who received famciclovir 2 hours before food. When famciclovir 250 mg was administered 30 minutes after food, $C_{max}$ was reduced by only 20%.

### SIDE EFFECTS AND INTERACTIONS

Tolerability data from trials of famciclovir for the treatment of genital herpes and herpes zoster indicate that famciclovir is well tolerated with an adverse event profile similar to placebo.[12,18] In an analysis of several trials, side effects did not correlate with increasing daily dose over a range of 125 mg/day to 2,250 mg/day. The most frequent adverse events reported in this analysis were headache, nausea, and diarrhea, which occurred in >2% of patients in both famciclovir and placebo groups. Serious adverse events were no more frequent than placebo and none were classified as related to or probably related to famciclovir.[17]

In a recent dose-finding trial of twice-daily oral famciclovir for recurrent genital herpes,[19] there were no significant differences in adverse events or withdrawals between the famciclovir (125, 250, and 500 mg twice-daily) and placebo groups. Most frequent adverse effects reported for both groups were headache, nausea, and dizziness.

A recent double-blind placebo-controlled study in which 34 men with recurrent genital herpes received famciclovir 250 mg twice daily for 18 weeks reported that famciclovir did not appear to have significant effects on any of the sperm parameters measured.[20]

No clinically significant alterations in penciclovir pharmacokinetics were observed following single-dose administration of 500 mg famciclovir after pre-treatment with multiple doses of cimetidine, allopurinol, or theophylline.[6]

### SPECTRUM OF ANTIVIRAL ACTIVITY

Famciclovir is effective against HSV-1, HSV-2, VCV, and the Epstein-Barr virus (EBV). Penciclovir has also been shown to inhibit viral replication in vitro in duck hepatocytes infected with duck hepatitis B virus (HBV)[12,21] and famciclovir has been shown to inhibit HBV replication in vivo in hepatic and nonhepatic tissue of ducklings that had been infected in ovo with duck HBV.[22]

### CLINICAL APPLICATIONS

Famciclovir is currently licensed to treat VZV herpes zoster (shingles) and HSV genital herpes infections.

Two large clinical trials evaluated famciclovir's effect on rash healing of acute herpes zoster and control of acute pain. A prospective, randomized, placebo-controlled, double-blind trial evaluated famciclovir (500 or 750 mg three times daily for 7 days) in 419 intent-to-treat immunocompetent patients with herpes zoster rash.[23] Famciclovir accelerated the healing of skin lesions (reduced times to full crusting and loss of vesicles, ulcers, and crusts) compared to those given placebo and significantly reduced duration of viral shedding and pain. More importantly, patients receiving famciclovir lost post-herpetic neuralgia (PHN; defined as pain persisting after lesion healing) a median of approximately 60 days sooner than those on placebo.

A double-blind, double-dummy, randomized study compared the safety and efficacy of famciclovir (250, 500, or 750 mg three times a day) and aciclovir (800 mg five times a day) for 7 days in 545 immunocompetent patients with acute herpes zoster.[24] For the intent-to-treat population, all doses of famciclovir were equally effective as aciclovir for healing lesions and shortening the time to loss of acute pain. With early treatment, there was approximately 1.5–1.8-fold acceleration in loss of pain over aciclovir, reinforcing the potential benefit of early treatment.

To determine the efficacy and safety of famciclovir in the episodic treatment of recurrent genital herpes, a multicenter, randomized, double-blind, dose-ranging, patient-initiated study was conducted comparing three twice-daily doses (125, 250, and 500 mg) of oral famciclovir with placebo over a 5-day period.[18] Famciclovir at all doses was significantly more effective than placebo at reducing time to healing, time to cessation of viral shedding, as well as durations of lesion edema, vesicles, ulcer, and crusts. Times to cessation of all symptoms and of moderate to severe lesion tenderness, pain, and burning were also reduced. Patients who initiated famciclovir prior to viral shedding more frequently aborted the onset of viral shedding. All doses were equally effective and well tolerated.

A similar multicentre, clinic-initiated trial using oral famciclovir at the same three doses again demonstrated reductions of viral shedding, lesion discomfort, lesion duration, and new lesion formation in famciclovir-treated (all doses) patients.[25] Lesion tenderness, itching, edema, and ulcer duration were also significantly reduced, and culture positive new lesions were more frequently avoided.

Recent trials have also shown favourable results with famciclovir for both the treatment of first episode genital herpes and for the suppression of recurrent genital herpes,[26] although these are not currently licensed indications in North America. Optimal dosing of famciclovir for first episodes was 250 mg tid, while for suppression, optimal dosing was 250 mg bid, and while for suppression, optimal dosing was 250 mg bid.[27]

In a double-blind, placebo-controlled pilot study of 17 patients with chronic HBV infection, famciclovir (250 mg or 500 mg three times daily) was found to cause a 90% decrease in levels of HBV-DNA in six patients on the drug.[28] This effect was maintained in four patients throughout the 10-day treatment period. Famciclovir alone,[29,30] or in combination with prostaglandin E,[31] has also been used successfully to suppress HBV replication in liver transplant patients. Further studies are required in HBV, however, before clinical indications, if any, can be determined.

TABLE I. Costs of selected antiviral agents (in U.S. dollars)

| Product | Dose | Cost/ dose | Tablets/ course of therapy | Cost of therapy |
|---|---|---|---|---|
| Zoster | | | | |
| Famvir | 500 mg tid/ 7 days | $4.92 | 21 | $103.32 |
| Aciclovir | 800 mg 5×/day | | | |
| | 7 days: | $3.41 | 35 | $119.35 |
| | 10 days: | $3.41 | 50 | $170.50 |
| Valaciclovir | 1000 mg tid/7 day | $4.60 | 42 | $96.60 |
| Recurrent GH | | | | |
| Famciclovir | 125 mg bid/ 5 days | $1.85 | 10 | $18.50 |
| Aciclovir | 200 mg 5×/day 5 days | $0.90 | 25 | $22.50 |
| | 400 mg tid/ 5 days[a] | $1.76 | 15 | $26.40 |
| Valaciclovir | 500 mg bid/ 5 days | $2.30 | 10 | $23.00 |
| 1st Episode GH | | | | |
| Famciclovir (under FDA review) | 250 mg tid/ 5 days | $2.45 | 15 | $36.75 |
| Aciclovir | 200 mg 5×/day/ 10 days | $0.90 | 50 | $45.00 |
| | 800 mg tid 7 days[a] | $3.41 | 21 | $71.61 |
| Valaciclovir (under FDA review) | 1000 mg bid/5 days | $4.60 | 20 | $46.00 |

[a]Alternative CDC recommended dose/non FDA approved.

### COST

Table 1 presents the comparative costs to the patient in the US for the antivirals famciclovir, aciclovir, and valaciclovir.

### ACKNOWLEDGMENTS

I would like to thank Bruce R. Wilson for his assistance with preparation of this manuscript.

### REFERENCES

1. Vere Hodge RA, Sutton D, Boyd MR, Harnden MR, Jarvest RL: Selection of an oral prodrug (BRL 42810; Famciclovir) for the antiherpesvirus agent BRL 39123 [9-(4-hydroxy-3-hydroxymethylbut-1-yl)guanine; penciclovir]. Antimicrob Agents Chemother 33:1765–1773, 1989.
2. Vere Hodge RA, Cheng Y-C: The mode of action of penciclovir. Antiviral Chem Chemother 4:13–24, 1993.
3. Perry CM, Wagstaff AJ. Famciclovir: A review of its pharmacological properties and therapeutic efficacy in herpesvirus infections. Drugs 50:396–415, 1995.
4. Pue MA, Benet LZ: Pharmacokinetics of famciclovir in man. Antiviral Chem Chemother 4:47–55, 1993.
5. Filer CW, Allen GD, Brown TA, et al.: Metabolic and pharmacokinetic studies following oral administration of $^{14}C$-famciclovir to healthy subjects. Xenobiotica 24:357–368, 1994.
6. Famvir® Product Brochure. SmithKline Beecham Pharmaceuticals.
7. Weller S, Blum R, Doucette M, et al.: Pharmacokinetics of the acyclovir pro-drug valaciclovir after escalating single and multiple-dose administration to normal volunteers. Clin Pharmacol Ther 54:595–605, 1993.
8. Vere Hodge RA: Famciclovir and penciclovir: The mode of action of famciclovir including its conversion to penciclovir. Antiviral Chem Chemother 4:67–84, 1993.
9. Gnann Jr JW: New antivirals with activity against varicella-zoster virus. Ann Neurol 34:569–72, 1994.
10. Winton CF, Fowles SE, Vere Hodge RA, et al.: Assay of famciclovir and its metabolites, including the antiherpes agent penciclovir, in plasma and urine of rats, dog, and man. In Reid E, Wilson ID (eds): Analysis of Drugs and Metabolites. Cambridge: Royal Soc Chemistry, pp 163–71, 1990.
11. Fowles SE, Pue MA, Pierce D, et al.: Pharmacokinetics of penciclovir in healthy elderly subjects following a single oral administration of 750 mg famciclovir [abstract]. Br J Clin Pharmacol 34:450P, Nov. 1992.
12. Cirelli R, Herne K, McCrary M, Lee P, Tyring SK: Famciclovir: Review of clinical efficacy and safety. Antiviral Res 29:141 151, 1996.
13. Boike SC, Pue MA, Freed MI, et al.: Pharmacokinetics of famciclovir in subjects with varying degrees of renal impairment. Clin Pharmacol Ther 55:418–26, Apr. 1994.
14. Boike SC, Pue M, Audet PR, et al.: Pharmacokinetics of famciclovir in subjects with chronic hepatic disease. J Clin Pharmacol 34:199–207, 1994.
15. Fowles SE, Fairless AJ, Pierce DM, et al.: A further study of the effect of food on the bioavailability and pharmacokinetics of penciclovir after oral administration of famciclovir [abstract]. Br J Clin Pharmacol 32:657P, Nov. 1991.
16. Fowles SE, Pierce DM, Prince WT, et al.: Effect of food on bioavailability and pharmacokinetics of penciclovir, a novel antiherpes agent, following oral adminstration of the pro-drug, famciclovir [abstract]. Br J Clin Pharmacol 29:620P–1P, May 1990.
17. Pratt SK, Standring-Cox R, Writer D, et al.: Penciclovir pharmacokinetics in fed and fasted subjects following oral famciclovir in relation to in-vitro antiviral activity [abstract]. Proc 6th International Congress for Infectious Diseases (ICID). April 26–30, 1994.
18. Saltzman R, Jurewicz R, Boon R: Safety of famciclovir in patients with herpes zoster and genital herpes. Antimicrob Agents Chemother 38:2454–2457, 1994.
19. Sacks SL, Aoki FY, Diaz-Mitoma F, Sellors J, Shafran SD, for the Canadian Famciclovir Study Group: Patient-initiated, twice-daily oral famciclovir for early recurrent genital herpes: A randomized, double-blind multicenter trial. JAMA 276:44–49, 1996.
20. Sacks SL, Bishop AM, Fox R, et al.: A double-blind, placebo (PLB)-controlled trial of the effect of chronically administered oral famciclovir (FCV; BRL42810) on sperm production in men with recurrent genital herpes (RGH) infection [abstract]. Antiviral Res 23:72, 1994.
21. Shaw T, Amor P, Civitico G, Boyd M, Locarnini S: In vitro activity of penciclovir, a novel purine nucleoside, against duck hepatitis B virus. Antimicrob. Agents Chemother 38:719–723, 1994.
22. Tsiquaye KN, Slomka MJ, Maung M: Famciclovir against duck hepatitis B virus replication in hepatic and nonhepatic tissues of ducklings infected in ovo. J Med Virol 42:306–310, 1994.
23. Tyring S, Barbarash RA, Nahlik JE, Cunningham A, Marley J, Heng M, Jones T, Rea T, Boon R, Saltzman R, and the Collaborative Famciclovir Herpes Zoster Study Group: Famciclovir for the treatment of acute herpes zoster: Effects on acute disease and posherpetic neuralgia. Ann Intern Med 123:89–96, 1995.
24. Degreef H. and the Famciclovir Herpes Zoster Clinical Study Group: Famciclovir, a new oral antiherpes drug: Results of the first controlled clinical study demonstrating its efficacy and safety in the treatment of uncomplicated herpes zoster in immunocompetent patients. Int J Antimicrob Agents 4:241–246, 1994.
25. Sacks SL, Martel AY, Aoki FY, Shafran SD, St.-Pierre C, Lassonde M, and the Canadian Cooperative Study Group: Early, Clinic-Initiated Treatment of Recurrent Genital Herpes Using Oral Famciclovir: Results of a Canadian, Multicentre Study. Clinical Research Meeting, American Federation of Clinical Research 1994, Baltimore, Maryland (abstract).
26. Mertz GJ, Loveless MO, Kraus SJ, Tyring SK, Fowler SL, and the Collaborative Famciclovir Genital Herpes Research Group: Famciclovir for Suppression of Recurrent Genital Herpes. Interscience Conference on Antimicrobial Agents and Chemotherapy 1995, 11:H3 (abstract).
27. Diaz-Mitoma F, Sibbald RG, Shafran SD, and the Collaborative Famciclovir Genital Herpes Suppression Group: Famciclovir suppression of recurrent genital herpes. (abstract). Interscience Conference on Antimicrobial Agents and Chemotherapy 1996, New Orleans, LA
28. Main J, Brown JL, Karayiannis P, et al.: A double-blind, placebo-controlled study to assess the effect of famciclovir on virus replication in patients with chronic hepatitis B infection. J Hepatol 21:S32, 1994.

29. Angus P, Richards M, Bowden S, Ireton J, Jones R, Locarnini S: Succesful treatment of post liver transplant recurrent hepatitis B. Presented at 34th Interscience Conf. Antimicrobial Agents and Chemotherapy (ICCAC), Oct. 94, Orlando, FL.
30. Angus P, Neuhaus P, Manns MP, and the Famciclovir Liver Transplant Group: An open study to assess the effect of famciclovir on hepatitis B replication in patients who have received an orthotopic liver transplant. Presented at Liver Transplantation for Chronic Viral Hepatitis, Mar. 1995, Reston VA.
31. Boker KHW, Ringe B, Froger M, Picelmayr R, Manns MP: Prostaglandin E plus famciclovir: A new concept for the treatment of severe hepatitis B after liver transplantation. Transplanation 57:1706–1708, 1994.
